# The Impact of Preterm Birth on Parents’ Mental Health and the Role of Family-Centred Interventions: A Narrative Review

**DOI:** 10.3390/children12101311

**Published:** 2025-09-29

**Authors:** Dora Mihaela Cîmpian, Gabriela Elena Strete, Cristian Ioan Cîmpian, Laura Mihaela Suciu, Manuela Cucerea, Vladimir Bacârea, Lucian Pușcașiu

**Affiliations:** 1 Doctoral School of Faculty of Medicine, George Emil Palade University of Medicine, Pharmacy, Science and Technology of Târgu Mureș, 540139 Târgu Mureș, Romania; dora.cimpian@umfst.ro; 2 Department of Psychiatry, George Emil Palade University of Medicine, Pharmacy, Science and Technology of Târgu Mureș, 540139 Târgu Mureș, Romania; 3 First Obstetrics and Gynecology Clinic, George Emil Palade University of Medicine, Pharmacy, Science and Technology of Târgu Mureș, Gheorghe Marinescu Street, Number 38, 540142 Târgu Mureș, Romania; cristicimpian@yahoo.com; 4 Department of Pediatrics 4, George Emil Palade University of Medicine, Pharmacy, Science and Technology of Târgu Mureș, Gheorghe Marinescu Street, No. 38, 540139 Târgu Mureș, Romania; laura.suciu@umfst.ro; 5 Department of Neonatology, George Emil Palade University of Medicine, Pharmacy, Science and Technology of Târgu Mureș, 540142 Târgu Mureș, Romania; manuela.cucerea@umfst.ro; 6 Department M2, Research Methodology, George Emil Palade University of Medicine, Pharmacy, Science and Technology of Târgu Mureș, 540139 Târgu Mureş, Romania; vladimir.bacarea@umfst.ro; 7 Departament of Obstretics and Gynecology, George Emil Palade University of Medicine, Pharmacy, Science and Technology of Târgu Mureș, 540139 Târgu Mureș, Romania; lucian.puscasiu@umfst.ro

**Keywords:** preterm birth, postpartum depression, parental stress, FCC, FiCare

## Abstract

Background/Objectives: Preterm birth is defined by the World Health Organization (WHO) as birth occurring before 37 weeks of gestation and represents one of the major public health concerns worldwide. Approximately 15 million newborns are affected annually. Following such a physically and emotionally traumatic event, most parents experience emotional distress and seek answers regarding the possible internal or external triggers. The main objective of this review is to analyze the current data regarding the impact of prematurity on parental mental health, as well as the types of interventions targeting parents. Methods: This narrative review was conducted based on extensive research of full-text scientific articles published in the past 15 years, investigating the relationship between prematurity, neonatal intensive care unit (NICU) hospitalization, parental mental health, and proposed intervention strategies aimed at supporting families. Results: Approximately 35% of mothers of preterm infants presented postpartum depression, 24% anxiety, and 15% PTSD. FCC interventions reduced stress levels and the intensity of depressive symptoms, while FICare showed stronger benefits, with additional improvements in parental mental health, parental self-efficacy, increased parental confidence, and amelioration of preterm infant parameters. Conclusions: Implementing FCC and FICare into daily neonatal care is essential for the prevention of parental mental health disorders and strengthening parenting capacity.

## 1. Introduction

Preterm birth or prematurity is defined by the World Health Organization (WHO) as birth occurring before 37 weeks of gestation and represents one of the major public health concerns worldwide. According to the WHO, approximately 13.4 million babies were born preterm in 2020. The prevalence varies between 4% and 16% depending on the country and region [1]. Recent medical advances in the field of neonatology have led to a significant increase in the survival rates of newborns with extremely low gestational age and birth weight in recent years. Nevertheless, this remarkable progress continues to be marked by complex challenges for both the affected families and the healthcare system [1,2].

Preterm birth at a very early gestational age is a profoundly stressful event for both parents. Hospitalization in a neonatal intensive care unit (NICU) creates an extremely demanding environment, characterized by the physical separation of the newborn from the parents, uncertainty regarding prognosis, and restricted direct interactions [2,3,4]. Severe maternal complications may also occur in the context of preterm birth, including hemorrhage, infections, and pulmonary or cardiovascular disorders, which may require the admission of both the newborn and the mother to intensive care units. These events can cause substantial psychological distress within the first hours after delivery, driven both by the infant’s medical condition and the abrupt adaptation to the parental role in a highly medicalized context [2,5,6].

Following such a physically and emotionally traumatic event, most mothers seek answers regarding the precipitating causes and, not infrequently, experience self-blame and ruminative thinking about possible internal or external triggers. The risk of developing secondary mental health disorders—such as anxiety, depression, and post-traumatic stress disorder (PTSD)—is elevated for both mothers and fathers in this context. In terms of the couple’s relationship, quality is generally not affected immediately following preterm birth; however, potential negative implications may emerge over the long term, particularly if the event impacts the child’s developmental trajectory [1,7,8,9,10].

Numerous studies have demonstrated that the NICU experience can precipitate affective disorders, including postpartum depression, generalized anxiety, and post-traumatic stress symptoms, with a higher prevalence than among parents of term-born infants [5,11,12]. The impact is not confined to the hospitalization period—symptoms may persist for months or even years, influencing parent–child relationships and the child’s psychosocial development [5,13].

In recent years, several family-focused interventions have been implemented, most notably the Family-Centred Care (FCC) and Family Integrated Care (FICare) models. Research has demonstrated the effectiveness of these approaches and the urgent need to adapt them to each country’s socio-cultural and economic context [14,15,16,17,18]. The presence of both mother and father during critical periods has been shown to be beneficial in numerous studies conducted in both the United States and Europe [14,15,16,17,18]. These models promote active parental participation in daily care procedures, open communication with the medical team, multidisciplinary collaboration, and personalized psychological support tailored to the family—aimed at reducing parental stress and improving neonatal outcomes [11,14,15,19,20].

The primary objective of this review is to analyze recent scientific literature on the impact of prematurity and NICU hospitalization on parental mental health, as well as the types of interventions implemented to support parents, with particular emphasis on psychological and family-centred strategies in crisis situations.

## 2. Materials and Methods

This review was conducted based on extensive research of full-text scientific articles published in the past 15 years, investigating the relationship between prematurity, neonatal intensive care unit (NICU) hospitalization, parental mental health, and proposed intervention strategies aimed at supporting families. The sources include observational studies, multicenter randomized clinical trials, systematic reviews, and meta-analyses.

The literature search was performed in the PubMed and Google Scholar databases. Combinations of keywords were used, such as “preterm infant, prematurity, preterm birth, neonatal intensive care unit, NICU, parental mental health, mental disorders, postpartum depression, anxiety, acute stress disorder, post-traumatic stress disorder, Family-Centred Care, Family Integrated Care, FCC, FICare”.

Inclusion criteria comprised studies published in internationally indexed journals, between 2012 and 2025, with the target population being parents (mothers and/or fathers) of preterm infants hospitalized in the NICU. Exclusion criteria included studies conducted before 2012, preclinical studies, papers published in languages other than English, and results based on interventions other than Family-Centred Care, Family Integrated Care with its variants mobile Family Integrated Care and Family Integrated Care Plus. Relevant data from each study were extracted manually and included the following: participant characteristics, intervention context, type and duration of psychological intervention, and the assessment tools used to evaluate psychological and mental health parameters.

## 3. Results

In total, 65 studies met the eligibility criteria and were included in this narrative synthesis. These comprised research addressing parental psychological outcomes associated with prematurity and NICU hospitalization, as well as studies describing family-centred or psychological interventions.

### 3.1. Prematurity and Postpartum Mental Disorders

#### 3.1.1. Risk Factors

Prematurity represents a major cause of neonatal morbidity and mortality, as well as a significant trigger for psychological disturbances in parents, particularly mothers. In many cases, preterm birth occurs suddenly, without adequate emotional preparation for the parents, leading to an abrupt transition from the anticipated course of pregnancy to the confrontation with a critical medical situation [1,2]. Studies published in the scientific literature report a high incidence of psychological disorders among parents experiencing the trauma of having a newborn admitted to a neonatal intensive care unit (NICU) [2]. Based on gestational age at delivery, preterm birth is classified into the following: moderate to late preterm (32–36 completed weeks of gestation), very preterm (before 32 completed weeks of gestation), and extremely preterm (before 28 completed weeks of gestation) [2,21].

Multiple antenatal and postnatal maternal risk factors have been identified that may increase the likelihood of preterm birth and, indirectly, contribute to the development of subsequent psychological disorders. These include advanced maternal age at first pregnancy, obesity, diabetes, chronic arterial hypertension, pre-existing dyslipidemia, substance use disorders (alcohol, tobacco, or illicit drugs), gestational diabetes, severe preeclampsia, cerebrovascular accidents, cardiac complications, and other life-threatening conditions [22,23,24,25,26,27,28].

A review of meta-analytic studies conducted by Mitrogiannis et al. identified approximately 166 risk factors for preterm birth [29]. The following risk factors with strong epidemiological credibility were highlighted: amphetamine exposure, maternal personality disorders, sleep-disordered breathing, reduced gestational weight gain, and an interpregnancy interval of less than 6 months following miscarriage [29]. Maternal psychosocial risk factors that may also be associated with preterm birth include homelessness, marital status, unintended pregnancy, and childhood abuse or trauma [30]. These maternal risk factors for preterm birth may exacerbate maternal psychological vulnerability, influencing postpartum mental health in the context of NICU hospitalization.

#### 3.1.2. Mechanism

Literature-based findings:

The pre-existence of maternal complications and comorbidities associated with psychological disturbance following preterm birth are associated with higher levels of maternal stress [2,5,31]. Physical separation and disruption of the parental role are also key factors, often manifested as a perceived loss of control and parental identity, particularly during the first weeks postpartum [3,13]. Prognostic uncertainty and the fear of death or long-term complications contribute to high levels of chronic stress. This activates the hypothalamic–pituitary–adrenal axis, leading to adrenal hyperfunction and cortisol hypersecretion, which contribute to the pathophysiological mechanisms underlying mental health disorders [11,15,24,32].

The environment of neonatal intensive care units (NICUs) can represent an additional source of stress for the mothers and fathers of newborns. Contributing factors to elevated stress levels include visual and auditory stimuli, the infant’s behaviour and appearance, certain medical procedures performed on the child, as well as the parent’s perceived role and capacity for parenting at that time [31,33]. These aspects, along with the stress perceived by the parent, can be assessed using the PSS:NICU (Parental Stressor Scale: Neonatal Intensive Care Unit) [31,33,34]. Numerous studies have demonstrated heightened levels of stress and discomfort among parents—particularly mothers—of newborns in relation to these analyzed items. Stress correlates both with the duration of hospital stay associated with the number and severity of medical procedures performed on the infant [2,11,20,27,28,35,36].

Longitudinal studies reveal that, although stress levels tend to decrease after discharge, a substantial proportion of parents continue to exhibit symptoms of depression, anxiety, or post-traumatic stress disorder (PTSD) even 6–12 months postpartum [5,12,13,25]

**Figure 1:**
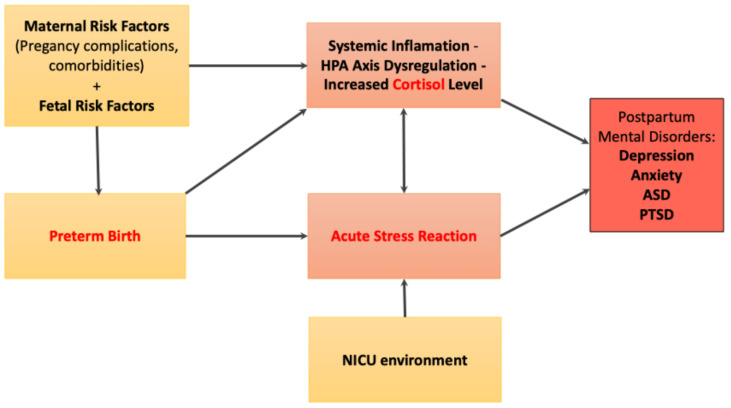
Conceptual model of the link between risk factors and postpartum maternal mental health.

Proposed conceptual model from preterm birth to postpartum maternal mental disorders:

Based on the existing literature [2,5,11,15,24,31,32], we propose the following conceptual model, as illustrated in Figure 1. Maternal and fetal risk factors predispose to preterm birth. Preterm delivery contributes to systemic inflammation through HPA axis dysregulation and elevated cortisol levels. In combination with the stress associated with the NICU environment, these biological and psychosocial factors can trigger an acute stress reaction, thereby increasing the risk of postpartum mental health disorders (Figure 1).

### 3.2. Postpartum Mental Disorders Associated with Preterm Birth

The parental experience of preterm birth, followed by the infant’s hospitalization in the neonatal intensive care unit (NICU), is associated with an increased prevalence of both immediate and long-term psychological disorders. The most frequent include depression, anxiety, acute stress reaction, and post-traumatic stress disorder (PTSD) [2,3,5,11,12,13,36].

#### 3.2.1. Postpartum Depression (PPD)

Postpartum depression among mothers of preterm infants has a prevalence of up to 35%, depending on the timing of the assessment and the measurement tools used [5,12,37,38,39]. Longitudinal studies indicate that depressive symptoms may appear early, within the first weeks after birth, and may persist up to one year postpartum [5]. Maternal risk factors include a personal history of affective disorders, antenatal comorbidities, and postpartum maternal complications [40]. External risk factors include low socioeconomic status and insufficient social support [11,12,41]. In a study conducted by Levinson M et al., it was demonstrated that higher scores on various items of the PSS:NICU scale were associated with increased odds of a positive screening for maternal postpartum depression. The intense presence of light and auditory stimuli was associated with higher PSS: NICU scores. The subjective perception of elevated stress increases the likelihood of obtaining a positive Edinburgh Postnatal Depression Scale (EPDS) score [42]. Depressive symptomatology has been evaluated in multiple studies using validated scales, such as the Edinburgh Postnatal Depression Scale (EPDS) [37] and the Nine-Item Patient Health Questionnaire (PHQ-9) [35,38]. However, it should be taken into account that these tools, EPDS and PHQ-9, are used as screening tests and not a definitive diagnosis for depression [35,37,38]. The prevalence, associated risk factors, and related symptom profiles are summarized in Table 1.

Several studies have reported an increased prevalence of maternal depressive symptoms associated with the severity of prematurity. Pace et al. found rates of 40–50% immediately postpartum among mothers of extremely preterm (EP) and very preterm (VP) infants, decreasing over time but remaining significantly higher than in the general population [19]. Hofheimer et al. reported a 13.5% prevalence of positive EPDS scores among mothers of very preterm infants (VP), with 42–46% of these women having a history of antepartum psychiatric disorders [37]. Positive screening scores were also correlated with prolonged NICU hospitalization [37]. Lotterman et al. identified a prevalence of 31.1% among mothers of moderate-to-late preterm infants (M/LP), with 21.1% persisting at six months postpartum [44].

#### 3.2.2. Anxiety

Anxiety represents a persistent worry about the infant’s survival, fear of complications, and hypervigilance in monitoring clinical signs and vitals [2,11,12,48]. Meta-analyses report a prevalence of clinically significant anxiety of approximately 24% among mothers and fathers, with the highest rates observed during the first 2–4 weeks of the infant’s hospitalization [12]. The Generalized Anxiety Disorder–7 Item Scale (GAD-7), State Trait Anxiety Inventory (STAI), and Perinatal Anxiety Screening Scale (PASS) are usually used for quantification. In a randomized study realized by Gensichen et al., a prevalence of up to 17% was reported for clinically manifesting symptomatology [38]. Anxiety is frequently associated with depressive symptoms [43] (Table 1).

The level of anxiety was correlated with the severity of prematurity in many studies. Pace et al. demonstrated an anxiety prevalence of approximately 47–48% among mothers and fathers of very preterm (VP) infants immediately after birth, with these symptoms persisting in up to 25% of parents at six months [19]. According to the research by Blanc et al., mothers of extremely preterm (EP) and very preterm (VP) infants showed a high prevalence of anxiety symptoms (83% state anxiety and 63.4% trait anxiety), with these rates being higher than those observed in mothers of term infants [49]. According to the study by Lotterman et al., the prevalence of clinical anxiety was 24.7% during NICU hospitalization and 27.6% at six months postpartum among mothers of moderate- and late-preterm (M/LP) infants [44].

A review conducted by Garg et al. concluded that anxiety was significantly higher among mothers of preterm births aged between 19 and 24 years with a low socioeconomic status [50]. Similarly to depression, anxiety scores were significantly higher in mothers compared to fathers. The presence of visual and auditory stimuli in the NICU, as well as the lack of maternal involvement in the care of the newborn, have also been shown to increase the severity of anxiety [51].

#### 3.2.3. Acute Stress Disorder (ASD) and Post-Traumatic Stress Disorder (PTSD)

According to the Diagnostic and Statistical Manual of Mental Disorders, Fifth Edition (DSM-5), acute stress disorder develops within three days of a traumatic event experienced directly or indirectly and may persist for up to one month [48]. The principal symptomatology include avoidance behaviours, flashbacks, nightmares, intrusive thoughts about the event, anxiety, dissociative elements, and cognitive disturbances affecting attention and memory [38,43,48]. Preterm birth and related complications constitute significant psychological trauma. Studies have found acute stress disorder (ASD) in up to 28% of parents affected by preterm birth in general [43] (Table 1).

If the symptoms of ASD persist beyond one month, a diagnosis of PTSD is established [43]. According to a perspective study conducted by Wile et al., the prevalence of PTSD-related symptoms among parents in this context is up to 15% [44] (Table 1). PTSD symptomatology has been analyzed in relation to the severity of prematurity in numerous studies. In a study conducted by Schecter et al., it was found that PTSD-related symptoms were present in parents of both extremely preterm (EP) and late preterm infants (LP). It was demonstrated that the severity of PTSD was not correlated with the actual clinical severity of the newborn, but rather with the subjective severity perceived by the parents [52]. According to Pace et al., approximately 23–27% of parents of very preterm (VP) infants manifested clinically significant PTSD symptoms after birth, with rates remaining elevated at around 14–15% six months postpartum [19]. In a study conducted by Lotterman et al., approximately 15–16% of mothers of moderate- and late-preterm (M/LP) infants show clinically significant PTSD symptoms during NICU hospitalization and at six months postpartum, with consistently higher rates compared to mothers of full-term infants [44].

Among the most traumatic experiences reported by the parents are daily separation, lack of physical contact, IV/feeding tubes, witnessing painful procedures, interactions with healthcare staff, long-term uncertainty, and infant’s small-size [52]. Furthermore, the presence of PTSD symptoms was also observed approximately one year after the traumatic event of preterm birth [52].

### 3.3. Types of Interventions in the NICU Targeting Parents

Providing mental support to mothers and fathers in neonatal intensive care units (NICUs) is crucial for reducing stress, anxiety, and depression, as well as for managing PTSD and ASD symptoms. Such support also facilitates parents’ adaptation to their new role and strengthens the parent–infant relationship [6,11,15,40]. Interventions are categorized according to their protocols and principles (Table 2).

#### 3.3.1. Family-Centred Care (FCC)

Family-Centred Care (FCC) is a type of intervention that focuses on the family and parents, and is based on the following principles: respect and dignity, promotion of diversity, support in decision-making, flexibility, information sessions, multidisciplinary collaboration and communication, along with psychological and mental support through encouraging parents in the daily management of childcare. Moreover, it encourages and creates the appropriate framework for parents’ participation in medical visits, in the decision-making process, and for their access to neonatal intensive care units (NICUs) [14,15,16]. The implementation and application of these models have been demonstrated in multiple studies to reduce stress scores, alleviate symptoms of depression and anxiety, increase confidence in parental capacities and skills, and contribute significantly to the improvement of the parent–child relationship [11,14,55,56].

The reduction in stress levels in parents was demonstrated in the meta-analytic study conducted by Ding et al., which included 19 randomized controlled trials and highlighted the positive effects of Family-Centred Care (FCC) on parental mental health. The analyzed studies showed a significant reduction in PSS:NICU scores, which was associated with a significant decrease in parental anxiety and depressive symptoms [55].

Lower depression scores assessed by the EPDS were associated with higher FCCQ scores, as demonstrated in the study conducted by Axelin et al. [56]. Depressive symptoms had a prevalence of 25.3% among mothers and 8.3% among fathers at discharge, decreasing to 12.3% and 5.8% at 4 months, respectively. The impact of Family-Centred Care (FCC) significantly reduced the risk of depression, with each additional point in the FCC score being associated with a decrease in EPDS depression screening scores of 0.16–0.18 for mothers and 0.14–0.99 for fathers [56]. A gender-related difference was noted, with mothers reporting higher and more persistent depression scores compared with fathers. Key elements such as partnership with medical staff, emotional support, and involvement in decision-making were associated with a relevant decrease in the risk of depression and EPDS scores [56].

Parental satisfaction, self-confidence, and duration of neonatal hospitalization have been evaluated by Segers et al. The results showed that, in the NICU environment, parental satisfaction increased significantly when collaboration between staff and parents was strengthened, and active parental involvement in infant care was associated with a significant reduction in the length of stay. These findings underline the clear potential of FCC in enhancing parental skills and strengthening parents’ subjective confidence in their parenting capacity [57].

The need for mental support, the reduction in anxiety, and the focus on both parents experiencing the NICU were qualitatively demonstrated by the study conducted by Serlachius et al. Through this, the importance of developing and consistently implementing Family-Centred Care (FCC) in the NICU is highlighted. Parents described experiences of loss of control, rigid communication between the medical team and family, as well as the neglect of the paternal parent in relation to the NICU experience [58].

The impact on stress level, anxiety, and depression has been analyzed by Franck et al. Through this study, they showed that parents of children hospitalized in the NICU frequently present symptoms of anxiety, depression, and emotional distress. The implementation of Family-Centred Care (FCC) has been associated with the reduction in these psychological symptoms. However, the authors note that, despite consistent reports of decreased anxiety and depression in parents exposed to FCC interventions, the magnitude of the effect varies across studies [53].

#### 3.3.2. Family Integrated Care (FICare)

The implementation of the Family Integrated Care (FICare) model in neonatal intensive care units (NICUs) represents a family-centred approach, conceived and based on the structure of Family-Centred Care (FCC). In this model, parents are considered active members of the care team, being involved and trained by the medical personnel in the care and management protocol of newborns. This model includes rigorous training of parents in the care of the premature newborn, their direct involvement in daily procedures and clinical decision-making, as well as the provision of adequate mental and psychological support from the medical team. Studies conducted demonstrate that the FICare model approach is associated with significant benefits on the parents’ mental health [54,60].

The FICare model, described by Franck et al., elaborates and adapts the principles of Family-Centred Care by actively involving parents in the daily care of newborns in the NICU. This model is based on four key pillars: creating a parent-friendly environment, training the medical team, providing education and psychological support for parents, and ensuring their direct participation in care. The clinical studies described show that parents involved through the FICare model exhibit lower levels of anxiety and stress, greater confidence in their caregiving abilities, and a stronger emotional bond with their newborn [54]. Another pilot study conducted by O’Brien et al. highlights a positive impact on newborn parameters and on stress scores measured using the PSS:NICU, compared to the control group [61] (Table 3).

In a review research, Waddington et al. demonstrated the effectiveness of FICare implementation in several pilot studies and clinical trials. These have shown a significant reduction in parental stress, depression, and anxiety. An increased rate of natural feeding of newborns was also demonstrated in FICare-type models, associated with weight gain in premature infants, and a significant reduction in the length of hospitalization for newborns in these groups. These results support the implementation of the FICare model as an effective strategy for protecting parents’ mental health in the neonatal intensive care environment [59]. In a study conducted in China by Hei M et al., a reduction in the level of oxygen required for newborns in the NICU, a decrease in duration of hospitalizations, and a reduction in nosocomial infections was highlighted [62].

The effect on mental health of the mobile Family Integrated Care (mFICare) programme was analyzed by Franck et al. The effects of the Family-Centred Care (FCC) model were compared with an enhanced mobile protocol of FICare (mFICare) on maternal mental health after the discharge of preterm infants from the NICU (Table 3). Mothers who experienced high stress in the NICU and were included in mFICare showed fewer symptoms of depression and PTSD at discharge and at follow-up after discharge, compared with those in the FCC group [63].

The reduction in stress and anxiety levels was demonstrated by the cluster randomized study conducted by O’Brien et al. They compared groups that benefited from the implementation of the FICare model in neonatal intensive care units. The results demonstrate improvements in weight gain in preterm infants, PSS:NICU scores were lower in the group that benefited from FICare, and an increased frequency of natural breastfeeding was demonstrated compared to those who received standard care [64].

A pilot study conducted by Ansari et al. evaluated the feasibility and impact of an extended programme applied to critically ill preterm infants hospitalized in the NICU. The programme is an adaptation to these conditions and is called FICare Plus (Table 3). In order to assess the mental status of parents, validated scales were used such as the State-Trait Anxiety Inventory (STAI), the Parental Stressor Scale: NICU (PSS:NICU), and the Perceived Parenting Self-Efficacy Tool, useful for evaluating parental capacity. The results showed that parents in the FICare Plus group had significantly lower scores at discharge on the applied questionnaires, suggesting lower levels of anxiety and stress. In addition, they reported greater confidence in their parental abilities and increased dedication to the care of their preterm infants [65].

**Table 3:** Models of the Family Integrated Care (FICare) [54,59,63,65].

Model	Definition	Main Components	Parent Role
FICare (Family Integrated Care)	Extension of FCC where parents are part of the NICU team [54,59].	Parent-friendly environment, Healthcare team training Structured parental education Direct participation in care [54,59].	Active caregivers [54,59]
mFICare (Mobile-FICare)	Digital extension of FICare using mobile app tools [59,63]	Mobile education support Digital support groups Access to clinical rounds through technology [59,63]	Active caregivers—hybrid in-person and digital [59,63]
FICare Plus (Family Integrated Care Plus)	Adaptation of FICare for critically ill infants [65]	Parental involvement structured Support for complex medical cases [65]	Active caregivers in very critical status of infant [65]

## 4. Discussion

Preterm birth is widely recognized by the WHO as being not only a major cause of neonatal mortality and morbidity, but it is also considered an important and profound factor of mental and emotional stress for parents [1]. The sudden transition from a pregnancy with normal evolution to a premature event that involves intensive neonatal care represents a causal factor of mental vulnerability [2,38]. Studies conducted so far consistently report among parents, especially mothers, increased prevalence of postpartum depression (up to 35%) [43], anxiety (17–24%) [12,38], manifestations and symptoms from the spectrum of acute stress disorder (28–35%) [7,43] which can complicate with post-traumatic stress disorder (15% in mothers and 7% in fathers) [7]. These health problems are further aggravated by maternal risk factors, the severity of neonatal illness, prolonged hospitalization, and the interruption of the parental role through physical separation from the infant [7,11,38,43,46,47].

This need has led to the creation of care models that actively involve parents in the NICU environment and that provide appropriate psychological support. Family-Centred Care (FCC) is one of the pioneering approaches that takes parents into account. Within this model, emphasis is placed on the collaboration between the medical team and the parents of the preterm infant, permanent access to information is promoted, and parental participation in the therapeutic management regarding the care of the preterm infant is encouraged [11,53,55]. Statistical evidence resulting from studies conducted so far demonstrates that FCC is associated with reduced parental stress, decreased symptoms of depression and anxiety, and improved confidence in parenting abilities [53,55,57,58]. Large-scale studies (Axelin et al.) have demonstrated that higher FCCQ scores were associated with fewer depressive symptoms measured both at discharge and four months after the premature birth event [56]. In the same idea, a meta-analytic study (Ding et al.) confirmed the significant decreases in depression and anxiety symptoms associated with FCC-type interventions [55].

The qualitative study conducted by Serlachius et al. [58] highlights the necessity to reconceptualize the parental experience in neonatal intensive care units (NICUs) from a holistic and equitable perspective. Through interviews with parents in this context, the researchers identified frequent feelings of loss of control, rigid communication with the medical team, and, most concerning, a systematic neglect of the father within the caregiving and clinical communication process [58].

Family Integrated Care (FICare) represents a more structured and comprehensive model, which transforms parents into active members of the medical team that cares for the preterm infant. This protocol integrates the systematic education of parents associated with daily caregiving responsibilities and participation in medical rounds [53,54,59]. Not least, of significant importance is the emotional and psychological support offered by the medical team [53,54,59]. The specialized literature suggests that FICare-type models provide stronger mental benefits compared to FCC [59,60,63]. The study conducted by Waddington et al. reported significant reductions in anxiety and depressive symptoms in mothers included in FICare compared to the standard of the unit [59]. Franck et al. underline that mothers who experienced high levels of stress and who benefited from FICare presented fewer symptoms of depression and PTSD elements compared to those who benefited from the FCC model [63]. The importance for families whose cases are very critical was addressed through a specially designed programme called FICare Plus. Through this approach, the efficiency and safety of being applied to preterm infants in critical condition was demonstrated. FICare Plus reduced maternal anxiety at discharge, and improved the parental sense of self-efficacy and involvement in the care of the infant [65].

The available evidence in the current specialized literature suggests that, although FCC provides an important and effective baseline support, the more advanced integration sustained by FICare models ensures stronger and more targeted protection for parents’ mental health, including in critical neonatal conditions. These results underline the importance of moving from passive involvement to a real and active integration of parents in neonatal care. Most of the studies conducted have been carried out on mothers. It is important to emphasize the need for more analyses and studies involving the paternal parent in order to obtain complete and complex results regarding the mental status and intrafamilial dynamics in the long term after the discharge of preterm infants.

Although most results are promising, several limitations must be highlighted. First, there is remarkable heterogeneity regarding study design, intervention protocols, and outcome evaluation tools, which have been adapted to the socio-cultural context. This aspect makes a direct comparison of studies relatively difficult. Most of the evaluations are based on self-administered questionnaires with a relatively high level of subjectivity, which could increase the risk of bias. Moreover, most results come from high-income countries, which makes it difficult to extrapolate the findings to middle- and low-income countries. Last but not least, most studies have followed parents from a psychological perspective only until discharge or just a few months after discharge. This raises the challenge of extending studies in the long term, up to 1 year, in order to gather more realistic data regarding the evolution of parental mental status.

Future research should include more evaluations of fathers’ mental status and should include standardized protocols and evaluation systems in order to perform realistic comparisons. Studies that cover middle- and low-income countries are necessary to expand the research data.

## 5. Conclusions

The hospitalization of a preterm newborn in a neonatal intensive care unit (NICU) represents a substantial psychological and emotional event for parents, generating elevated levels of stress, anxiety, and depressive symptoms. These may have long-term consequences for the parent–child relationship and for parental mental health. Early family support interventions initiated during hospitalization can significantly reduce the severity of parental psychological symptoms and may have beneficial effects on the infant. Models of Family-Centred Care (FCC) and Family Integrated Care (FICare) have demonstrated their effectiveness in reducing stress, improving mental health status, and enhancing self-esteem and parenting capacity.

To provide adequate support for parental mental health, it is essential to integrate these care and family intervention protocols into standard neonatal practice, alongside adequate staff training, by promoting accessible and feasible programmes on a wider scale.

## Figures and Tables

**Table 1:** Summary of Postpartum Mental Disorders among parents associated with NICU experience [19,36,43,44,45,46].

Disorder	Prevalence	Onset	Risk Factors
Depression	-Up to 35% [43]	-first weeks postpartum -persist up to 1 year	-antecedents of affective disorders [7,43] -antenatal comorbidities [22] -maternal complications [22] -lack of socioeconomic support [11] -feelings of powerlessness [7]
Anxiety	-Up to 24% [12] -17% clinical symptoms [38]	-2–4 weeks on infant hospitalization	-preterm birth -comorbid with depression -fear of complications -high parental stress -primiparous mothers
Acute Stress Disorder (ASD)	-28–35% [7,43] mothers -up to 24% fathers [7]	-3 days to 1 month	-preterm birth [7,11], -neonatal complications [47] -maternal complications [47] -NICU traumatic experience and environment [7,11] -high parental stress level [11,43] -alteration of parental role
Post-Traumatic Stress Disorder (PTSD)	-Up to 15% mothers [7] -8% fathers [7]	-after 1 month	

**Table 2:** Comparison of the principles and effects of Family-Centred Care (FCC) and Family Integrated Care (FICare) in the NICU.

	Family-Centred Care (FCC)	Family Integrated Care (FICare)
Definition	A holistic model of care recognizing the family as a partner in the child’s healthcare [53].	Extension of FCC where parents are actively involved as part of the infant care team [53].
Main Principles	Respect and dignity Information sharing Inclusion Collaboration [53]	Healthy environment NICU team education and support Parental education and mental support Active participation/partnership in infant care [53]
Main Role of Parents	Supportive [53]	Active caregivers and co-participants [53]
Protocol	Passive participation [53] Involvement in care decisions [53] Open access to NICU [53] Mental and emotional support from medical team [53]	Participate in training sessions [54] Active participation in care routines [54] Integration in clinical decision [54]
Impact on Mental Health	Decrease stress level in parents [55] Reduce depressive symptoms [56] Increase parental satisfaction [57] Reduce anxiety [58]	Significantly reduce stress level [54] Significantly reduce anxiety [54] Significantly reduce depression [59] Improve parental self-esteem, efficacy, satisfaction, and bonding [54]

## Data Availability

This study is based on previously published literature. No new data were generated in this literature review, and data sharing is not applicable.

## Abbreviations

The following abbreviations are used in this manuscript:

ASD	Acute Stress Disorder
DSM-5	Diagnostic and Statistical Manual of Mental Disorders, Fifth Edition
EPDS	Edinburgh Postnatal Depression Scale
FCC	Family-Centred Care
FCCQ	Family-Centred Care Questionnaire
FiCare	Family Integrated Care
FICare Plus	Family Integrated Care Plus
GAD-7	Generalized Anxiety Disorder–7 Item Scale
HPA	Hypothalamic–Pituitary–Adrenal
mFICare	mobile Family Integrated Care
NICU	Neonatal Intensive Care Unit
PASS	Perinatal Anxiety Screening Scale
PHQ-9	Nine-Item Patient Health Questionnaire
PPD	Postpartum Depression
PSS:NICU	Parental Stressor Scale: Neonatal Intensive Care Unit
PTSD	Post-Traumatic Stress Disorder
STAI	State Trait Anxiety Inventory
WHO	World Health Organization
